# Knockdown of Ift88 in fibroblasts causes extracellular matrix remodeling and decreases conduction velocity in cardiomyocyte monolayers

**DOI:** 10.3389/fphys.2022.1057200

**Published:** 2022-11-17

**Authors:** Auriane C. Ernault, Makiri Kawasaki, Benedetta Fabrizi, Pablo Montañés-Agudo, Shirley C. M. Amersfoorth, Rushd F. M. Al-Shama, Ruben Coronel, Joris R. De Groot

**Affiliations:** Department of Clinical and Experimental Cardiology, Heart Center, Amsterdam Cardiovascular Sciences, Amsterdam UMC, University of Amsterdam, Amsterdam, Netherlands

**Keywords:** atrial fibrillation, fibrosis, fibroblasts, primary cilia, cardiac

## Abstract

**Background:** Atrial fibrosis plays an important role in the development and persistence of atrial fibrillation by promoting reentry. Primary cilia have been identified as a regulator of fibroblasts (FB) activation and extracellular matrix (ECM) deposition. We hypothesized that selective reduction of primary cilia causes increased fibrosis and facilitates reentry.

**Aim:** The aim of this study was to disrupt the formation of primary cilia in FB and examine its consequences on ECM and conduction in a co-culture system of cardiomyocytes (CM) and FB.

**Materials:** Using short interfering RNA (siRNA), we removed primary cilia in neonatal rat ventricular FB by reducing the expression of *Ift88* gene required for ciliary assembly. We co-cultured neonatal rat ventricular cardiomyocytes (CM) with FB previously transfected with *Ift88* siRNA (siIft88) or negative control siRNA (siNC) for 48 h. We examined the consequences of ciliated fibroblasts reduction on conduction and tissue remodeling by performing electrical mapping, microelectrode, and gene expression measurements.

**Results:** Transfection of FB with siIft88 resulted in a significant 60% and 30% reduction of relative *Ift88* expression in FB and CM-FB co-cultures, respectively, compared to siNC. Knockdown of *Ift88* significantly increased the expression of ECM genes *Fn1*, *Col1a1* and *Ctgf* by 38%, 30% and 18%, respectively, in comparison to transfection with siNC. Conduction velocity (CV) was significantly decreased in the siIft88 group in comparison to siNC [11.12 ± 4.27 cm/s (*n* = 10) vs. 17.00 ± 6.20 (*n* = 10) respectively, *p* < 0.05]. The fraction of sites with interelectrode activation block was larger in the siIft88 group than in the siNC group (6.59 × 10^−2^ ± 8.01 × 10^−2^ vs. 1.18 × 10^−2^ ± 3.72 × 10^−2^ respectively, *p* < 0.05). We documented spontaneous reentrant arrhythmias in two cultures in the siIft88 group and in none of the siNC group. Action potentials were not significantly different between siNC and siIft88 groups.

**Conclusion:** Disruption of cilia formation by siIft88 causes ECM remodeling and conduction abnormalities. Prevention of cilia loss could be a target for prevention of arrhythmias.

## Introduction

Atrial fibrillation (AF) is the most common arrhythmia, with an estimated 46.3 million individuals suffering from this condition worldwide (Kornej et al., 2020). AF not only significantly reduces the quality of life, but also results in an increased morbidity and mortality (Benjamin et al., 1998). AF is a progressive disease which starts as a non-sustained arrhythmia originating from ectopic activity, and often progresses to a sustained state *via* structural remodeling of the atria (Krogh-Madsen et al., 2012; Heijman et al., 2021).

Atrial fibrosis, the concert of proliferation of fibroblasts and differentiation into myofibroblasts and the pathological accumulation of extracellular matrix (ECM), is the main component of structural remodeling in AF (Nattel et al., 2008). Fibrosis causes heterogeneous conduction delay, facilitating the occurrence and maintenance of AF. Cardiac (myo) fibroblasts are the main cell population responsible for ECM production and fibrosis formation in AF. TGF-β1 and angiotensin II are the main drivers for cardiac fibroblasts to form such pathological accumulation of ECM (Burstein and Nattel, 2008).

Primary cilium is a small organelle that extends from a cell’s surface, and functions as a chemo- and mechano-sensors. Our proteome and transcriptome analysis of atrial tissue of patients with or without AF (non-AF) shows that the gene-sets of ciliogenesis, including *IFT88,* are down-regulated in AF compared to non-AF patients (Kawasaki et al., 2021; van den Berg et al., 2021). The dysregulation of primary cilia has been implicated in the fibrosis formation in various tissues, such as liver and kidney (Teves et al., 2019; Collins and Wann, 2020). Furthermore, our simultaneous study has indicated that the proportion of atrial fibroblasts with primary cilia is decreased in the left atrial tissue of persistent AF patients compared to non-AF patients, and the disruption of primary cilia in human atrial fibroblasts enhances their capacity to produce ECM. Thus, disruption of primary cilia in FB could activate them and cause accumulation of ECM, resulting in the formation of fibrosis in AF. However, it remains unclear what the direct consequences of FB with disrupted primary cilia are on cardiac conduction and action potential properties in multicellular preparations.

The aim of this study was to selectively decrease primary cilia in FB and examine its consequences on conduction and action potential properties in a co-culture system of cardiomyocytes (CM) and FB isolated from neonatal rats.

Here, we report that the selective disruption of primary cilia in FB by RNAi targeting *Ift88* induces ECM production, decreases conduction velocity, increases number of block lines and increases the risk or reentrant arrhythmias without changing the action potential (AP) characteristics of CM. Our data suggest that dysregulation of primary cilia causes fibrosis and hinders myocardial conduction, thereby facilitating the occurrence of re-entry and formation of the arrhythmogenic substrate.

## Materials and methods

### Neonatal rat ventricular myocytes and fibroblasts isolation

All animal experiments were approved by the local Animal Experiments Committee (Academic Medical Center, University of Amsterdam) and carried out in compliance with the Guide for the Care and Use of Laboratory Animals and in accordance with national and institutional guidelines. Neonatal rat ventricular CM and FB were isolated from 2-days-old Wistar rats (Janvier labs). Pups were anesthetized by isoflurane and hearts were excised after decapitation. Ventricles were cut in pieces and dissociated using trypsin (1 mg/ml; Sigma) and collagenase type 2 (1 mg/ml; Worthington). Cells were resuspended in TUNG culture medium (M199 medium, Gibco) supplemented with 10% heat inactivated fetal bovine serum (FB, Gibco), 1% HEPES (Gibco), 5,000 U/L penicillin-G (Sigma), 2 mg/L vitamin B12 (Sigma-Aldrich), 3.5 g/L glucose, 1% non-essential amino acids (Gibco), 1% L-glutamine (Gibco). The cell suspension was pre-plated for 2 h to physically separate the rapidly adhering FB from CM. CM in suspension were collected and plated on multi-electrode arrays (MEAs, Multi-Channel Systems MCS GmbH, Reutlingen, Germany) and on 24-well tissue culture plates coated with fibronectin (125 μg/ml BD Biosciences, Breda, Netherlands) at a density of 2.29 × 10^5^ cells per cm^2^. CM and FB were cultured at 37°C and 5% CO_2_.

### Transfection

In order to suppress the formation of primary cilia in FB, we downregulated the expression of the intraflagellar transport protein 88 gene (*Ift88*) required for ciliary assembly (Marshall, 2008). FB were transfected with 5 nM short interfering RNA (siRNA) for Ift88 (siIft88) (Silencer™ Select siIFT88, ThermoFisher Scientific, Cat#. 4390771, SiRNA ID. s157133) and with 5 nM negative control siRNA (siNC) (Silencer™ Negative Control, ThermoFisher Scientific, Cat#. AM4611) using Lipofectamine RNAiMAX reagent (Invitrogen, Cat#. 13778).

### Co-culture of fibroblasts and cardiomyocytes

Twenty-four hours after transfection, cells were digested with trypsin, collected and counted for co-culture experiments. FB were resuspended in 1 ml of TUNG culture medium and added to the CM culture plates/MEAs at a ratio of 1:0.27 (CM:FB). After 48 h of co-culture, cells were collected for RNA isolation. Electrical mapping and microelectrode measurements were also performed.

### qRT-PCR

Total RNA was extracted from co-cultured CM and FB with TRI reagent (Sigma-Aldrich) following manufacturer’s protocol. RNA was used to generate cDNA using Superscript II (Invitrogen). qRT-PCR was performed on a LightCycler 480 (Roche) with SYBR Green I Master (Roche). Results were analyzed using LinRegPCR software. Primer sequences are included in Table 1. Relative gene expression was calculated using reference gene *Hprt*. Gene expression in the siIFT88 experiments was normalized to siNC for each cell isolation, and expressed as fold change (FC).

**TABLE 1:** Primer sequences.

Primer	Sequence
COL1A1 Fw	CTG​AGC​CAG​CAG​ATC​GAG​AA
COL1A1 Rv	TCG​CTT​CCA​TAC​TCG​AAC​TGG
CTGF Fw	GCG​CCT​GTT​CTA​AGA​CCT​GT
CTGF Rv	TGC​ACT​TTT​TGC​CCT​TCT​TAA​TGT
GJA1 Fw	ACT​TCA​GCC​TCC​AAG​GAG​TTC
GJA1 Rv	GGT​GGA​GTA​GGC​TTG​GAC​CT
FN1 Fw	CCA​CCA​TCA​CTG​GTC​TGG​AG
FN1 Rv	GGG​TGT​GGA​AGG​GTA​ACC​AG
HPRT Fw	TGA​CTA​TAA​TGA​GCA​CTT​CAG​GGA​TTT
HPRT Rv	CGC​TGT​CTT​TTA​GGC​TTT​GTA​CTT​G
IFT88 Fw	CTG​GCA​GTG​ATA​GTG​GCC​AGA
IFT88 Rv	GCA​TTT​GCC​TAT​TTC​TTT​GTT​CCC
CACNA1G Fw	AGG​CAG​AGG​AAA​TCG​GCA​AA
CACNA1G Rv	CTG​TCC​CCA​TCA​CCA​TCC​AC
KCNJ11 Fw	ATC​AGT​CCA​GAG​GTT​GGT​GC
KCNJ11 Rv	TAA​TGC​CCT​TTC​GGG​ACA​GC
KCNQ1 Fw	GAT​CAG​TCC​ATC​GGG​AAG​CC
KCNQ1 Rv	GGT​CCA​GTT​GTG​TCA​CCT​TGT
KCNJ2 Fw	TGT​GTT​ACA​GAC​GAG​TGC​CC
KCNJ2 Rv	CAG​AGT​TTG​CCG​TCC​CTC​AT
SCN1B Fw	AAC​ACC​AGC​GTC​GTC​AAG​AA
SCN1B Rv	TTC​CGA​GGC​ATT​CTC​TTG​TGC
SCN5A Fw	TCT​TCC​GGT​TCA​GTG​CCA​CC
SCN5A Rv	GGA​TGG​TGC​ACA​TGA​TGA​GCA​TG

### Protein analysis

Protein were isolated using TRI reagent (Sigma-Aldrich) following manufacturer’s protocol.

Protein concentration was measured by Pierce™ BCA Protein Assay Kit (Thermo Scientific) following manufacturer’s instructions.

### Western blotting

Western blotting was performed following standard protocols. In short, proteins were separated by electrophoresis in 4%–15% Mini-PROTEAN TGX Precast Protein Gels (Bio-Rad) and transferred to PVDF membranes with the TransBlot Turbo Transfer System (Bio-Rad). Membranes were blocked in TBST 5% milk Protifar (Nutricia) for 1 h at room temperature, then they were incubated with the primary antibodies overnight at 4°C, and finally with HRP-conjugated secondary antibodies for 1 h at room temperature. Western blots were developed with ECL prime western blotting detection agent (Amersham Biosciences) in ImageQuant LAS 4000 (GE Healthcare Life Sciences). Western blots were quantified using Fiji (ImageJ). Antibody references are listed in Table 2.

**TABLE 2:** Antibodies.

Antibody	Dilution	References
Anti-connexin-43	1:1000 (Simple Wes) 1:250 (IF)	Sigma-Aldrich C6219
Anti-calnexin	1:250 (Simple Wes)	Sigma-Aldrich 208880
Anti-α-actinin	1:1000 (IF)	Sigma-Aldrich A7811
Anti-acetylated α-tubulin	1:1000 (IF)	Abcam 24610
Anti-vimentin	1:1000 (IF)	Abcam ab92547
Anti-IFT88	1:500 (WB)	Proteintech 13967-1-AP
Anti-GAPDH	1:10 000 (WB)	Fitzgerald 10R-G109A
Anti-rabbit-HRP	1:10 000 (WB)	Amersham NA9340V
Anti-mouse-HRP	1:10 000 (WB)	Amersham NA9310V

IF, immunofluorescence.

### Simple western analysis

Proteins were separated by size and detected with a WES system (ProteinSimple, San Jose, CA, United States) using a 12–230 kDa separation module with the primary and secondary antibodies mentioned in Table 2. Protein signal analysis and quantification was performed with the Compass software v.4.0.0 (Protein Simple).

### Immunofluorescence assays

Cells were fixed in 4% paraformaldehyde, washed and permeabilized (0.2% Triton X-100 in PBS), blocked with 4% goat serum and incubated overnight with the primary antibodies in a humidity chamber at 4°C. Coverslips were incubated for 1 h with secondary antibodies at room temperature, followed by DAPI (Molecular probes) staining. Images were acquired with a Leica DM6000B. The proportion of non-ciliated fibroblasts was quantified using ImageJ.

### Electrical mapping

Electrical mapping was performed as previously described (Ernault et al., 2022). Briefly, 10 min before measurements, CM-FB culture media were replaced by a modified Tyrode’s solution (36.5°C) containing (mM): NaCl 140, KCl 5.4, CaCl2 1.8, MgCl2 1.0, glucose 5.5, HEPES 5.0; pH 7.4. Spontaneous electrical activity was recorded from CM-FB co-cultured on MEAs (60 electrodes terminals) (60EcoMEA-Glass-gr, Multi Channel Systems MCS GmbH). A reference electrode was place inside the MEA, not in contact with the cells. Unipolar electrograms were recorded simultaneously with a 256-channel amplifier (BioSemi, 24 bit dynamic range, 122.07 nV LSB, total noise 0.5 μV). Signals were recorded with a sampling frequency of 2048 Hz [bandwidth (−3 dB) DC–400 Hz].

Data analysis were performed using a custom-made data analysis program written in Matlab 2006b [The MathWorks Inc, Natick, MA, United States (Potse et al., 2002)]. At each local unipolar electrogram, activation time (AT) was determined as the interval from a reference time zero to the minimum derivative of the QRS-complex of the local unipolar electrogram, and used to construct activation maps. Conduction velocity (CV) was determined along lines perpendicular to isochronal lines by dividing the distance by the difference in local AT. Number of block lines was assessed by counting the number of 30 ms block lines separating adjacent electrodes within one spontaneously active monolayer. The number of 30 ms block line was normalized to the total number of recording electrodes.

### Microelectrode measurements

Spontaneous APs were measured in CM-FB monolayers using glass pipette microelectrodes (Harvard apparatus GC100F- 10). These micro-electrodes were filled with 3M KCl, typical tip resistance was 15–25 MΩ. An AgCl-covered silver wire was used as a reference electrode. Resting membrane potential (RMP) was taken as the most negative membrane potential recorded at each recording position. Maximum AP upstroke velocity (V_max_) was measured and AP duration was determined at 40, 60, and 80% (APD_40_, APD_60_, APD_80_) of repolarization. All signal analyses were performed using a custom-made data analysis program written in Matlab 2006b [The MathWorks Inc, Natick, MA, United States (Potse et al., 2002)].

### Statistics

Statistical analyses were performed with GraphPad Prism (GraphPad Software, San Diego, CA, United States). Data are given as mean ± SD unless indicated otherwise. Number of observations and repeated experiments are given in the figure legends. Data were tested for normality using a Shapiro-Wilk normality test. If normal distribution was verified, the 2-tailed Student’s *t*-test was performed. If the data were not normally distributed, statistical significance was assessed using the 2-tailed Mann-Whitney test. *p* < 0.05 was considered significant.

## Results

### Knockdown of Ift88 in fibroblasts is associated with extracellular matrix production

**FIGURE 1:**
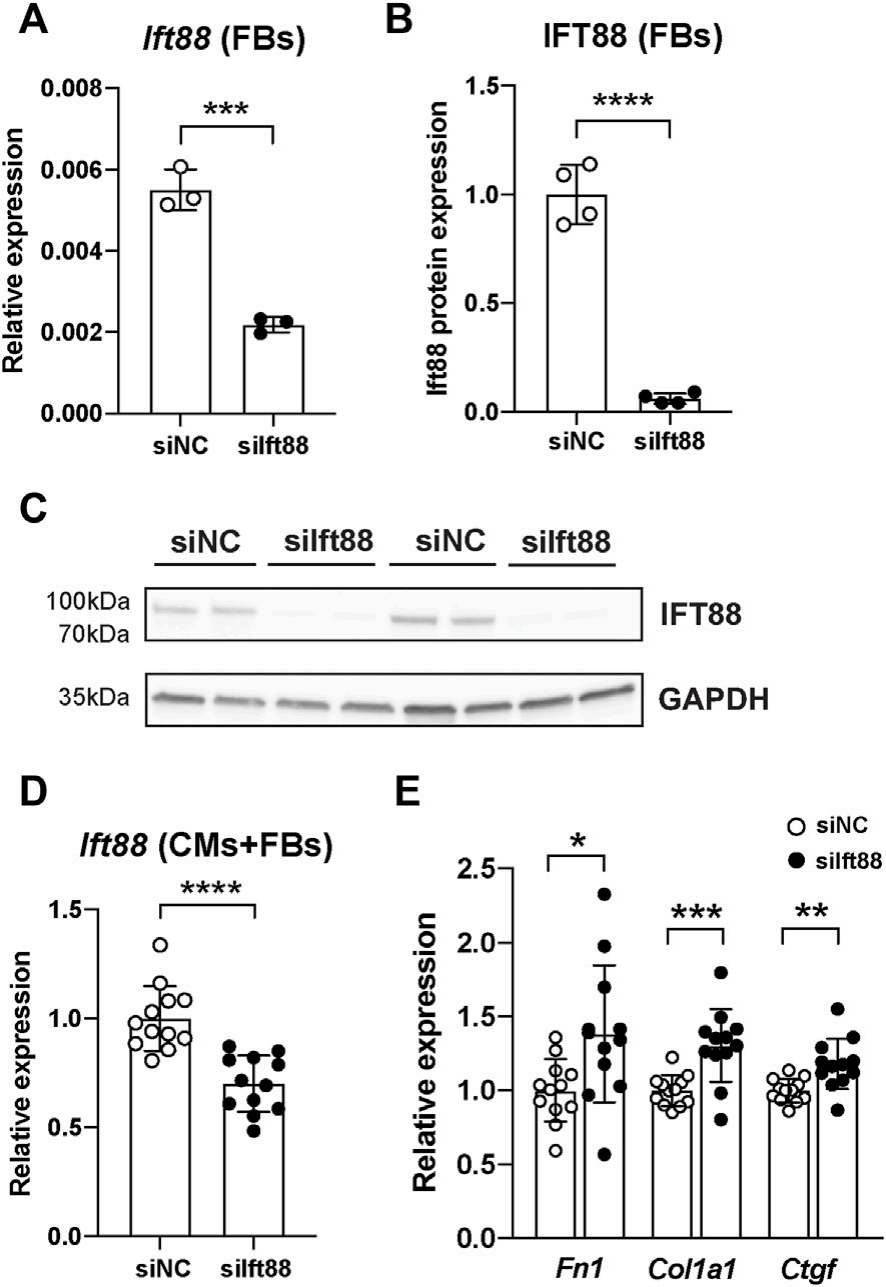
Reduction of *Ift88* expression in FB leads to increased ECM genes expression in CM and FB co-culture. **(A)** Relative expression of (mRNA) *Ift88* in FB previously transfected with siNC or siIft88. Data are mean ± SD. *n* = 6 from tree independent CM/FB isolation, Student’s *t*-test. **(B,C)** Relative expression of (protein) IFT88 in FB previously transfected with siNC or siIft88. Data are mean ± SD. *n* = 4 from two independent CM/FB isolation, Student’s *t*-test. Full unedited blots are provided in Supplementary Figure S1. **(D)** Relative expression of (mRNA) *Ift88* in CM-FB co-cultures after FB transfection with siNC or siIft88. Data are mean ± SD. *n* = 12 from four independent CM/FB isolation, Student’s *t*-test. **(E)** Relative expression of ECM genes *Fn1*, *Col1a1* and *Ctfg* after 48 h of co-culture of CM-FB. Data are mean ± SD. *n* = 12 from four independent CM/FB isolation, Student’s *t*-test. *p < 0.05, **p < 0.01, ***p < 0.001, ****p < 0.0001

To evaluate the knockdown efficiency of siIft88 in FB, we measured *Ift88* expression in FB only (mRNA and protein) and in CM-FB co-culture (mRNA). Transfection of FB with siIft88 resulted in a 60% significant reduction of (mRNA) *Ift88* expression in FB after 48 h of culture [2.18 × 10^−3^ ± 1.89 × 10^−4^ (siIft88) vs. 5.50 × 10^−3^ ± 4.99 × 10^−4^ (siNC), *p* < 0 .001, *n* = 6] in comparison to the group transfected with siNC (Figure 1A). In addition, transfection of FB with siIft88 resulted in a 94% significant reduction of IFT88 protein expression in FB [0.06 ± 0.02 (siIft88) vs. 1.0 ± 0.13 (siNC), *p* < 0 .0001, *n* = 8] in comparison to siNC (Figures 1B,C; Supplementary Figure S1). Furthermore, transfection of FB with siIft88 resulted in a 30% significant reduction of relative *Ift88* expression in the combined CM-FB co-culture, (FC = 0.07 ± 0.13, *p* < 0.0001, *n* = 12) in comparison to the group transfected with siNC (Figure 1B).

Next, we assessed whether *Ift88* reduction in fibroblasts was associated with reduction of primary cilia. Knockdown of *Ift88* in fibroblasts led to a significant increase in the proportion of non-ciliated fibroblasts in comparison to siNC (Supplementary Figure S2) [0.12 ± 0.03 (siIft88) vs. 0.07 ± 0.02 (siNC), *p* < 0.0001].

Knockdown of *Ift88* significantly increased the relative expression of ECM genes *Fn1*, *Col1a1* and *Ctgf* by 38%, 30% and 18%, respectively, in comparison to siNC (*Fn1*: FC = 1.38 ± 0.46, *p* < 0.05; *Col1a1*: FC = 1.30 ± 0.25, *p* < 0.001; *Ctgf*: FC = 1.18 ± 0.17, *p* < 0.01; *n* = 12) (Figure 1C).

### Co-culture of cardiomyocytes and fibroblasts with reduced cilia show heterogeneous conduction slowing and reentry

**FIGURE 2:**
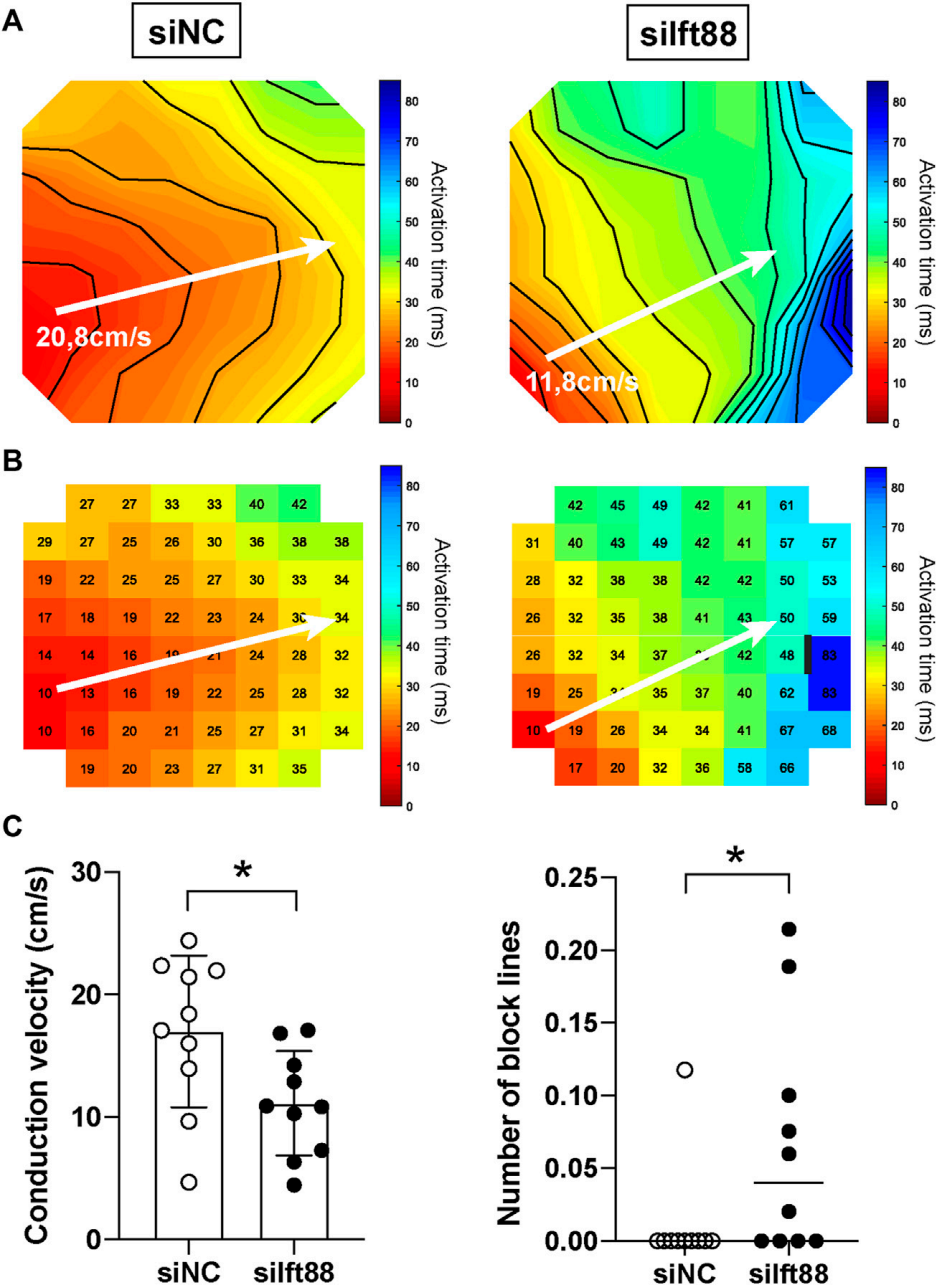
Reduced conduction velocity in CM monolayers co-cultured with decreased Ift88 FB. **(A,B)** Representative activation maps obtained after electrical mapping of spontaneously active CM co-cultured for 48 h with FB previously transfected with siNC or siIft88. Colors indicate activation times, according to the scale at right. Isochrones, 5 ms. White arrows indicate where conduction velocity was measured. Black line indicates 30 ms conduction block. Interelectrode distance of 700 μM. **(C)** Conduction velocity (CV) and number of block lines in spontaneously active CM monolayers co-cultured for 48 h with FB transfected with siNC or siIft88. Data are mean ± SD for CV and median for number of block lines, *n* = 10 monolayers from three independent CM/FB isolation. Student’s *t*-test for CV, Mann-Whitney test for number of block lines. Factor correction was carried out on CV to avoid inter-isolations differences.

To examine the effect of disrupted cilia formation in FB on conduction, we performed electrical mapping of CM co-cultured with FB pretreated with siIft88 or siNC. Activation maps generated from the siIft88 co-cultures showed isochrone crowding, revealing a slower conduction in siIft88 than in siNC co-cultures (Figures 2A,B).

On average, CV was 17.00 ± 6.20 cm/s in the siNC co-cultures, while it was 11.12 ± 4.27 cm/s in the siIft88 co-cultures (Figure 2C). CV was significantly lower in the siIft88 than in the siNC co-cultures (*p* < 0.05, *n* = 10).

Next, number of block lines was assessed by counting the number of 30 ms block lines in the activation maps, as shown in Figure 2B (right). Number of block lines was 1.18 × 10^−2^ ± 3.72 × 10^−2^ in the siNC co-cultures, while it was 6.59 × 10^−2^ ± 8.01 × 10^−2^ lines of block per electrode in the siIft88 co-cultures. Number of block lines was significantly higher in the siIft88 co-cultures than in the siNC (*p* < 0.05, *n* = 10).

### Cardiomyocytes monolayers co-cultured with fibroblasts with reduced cilia are more prone to reentrant arrhythmias

**FIGURE 3:**
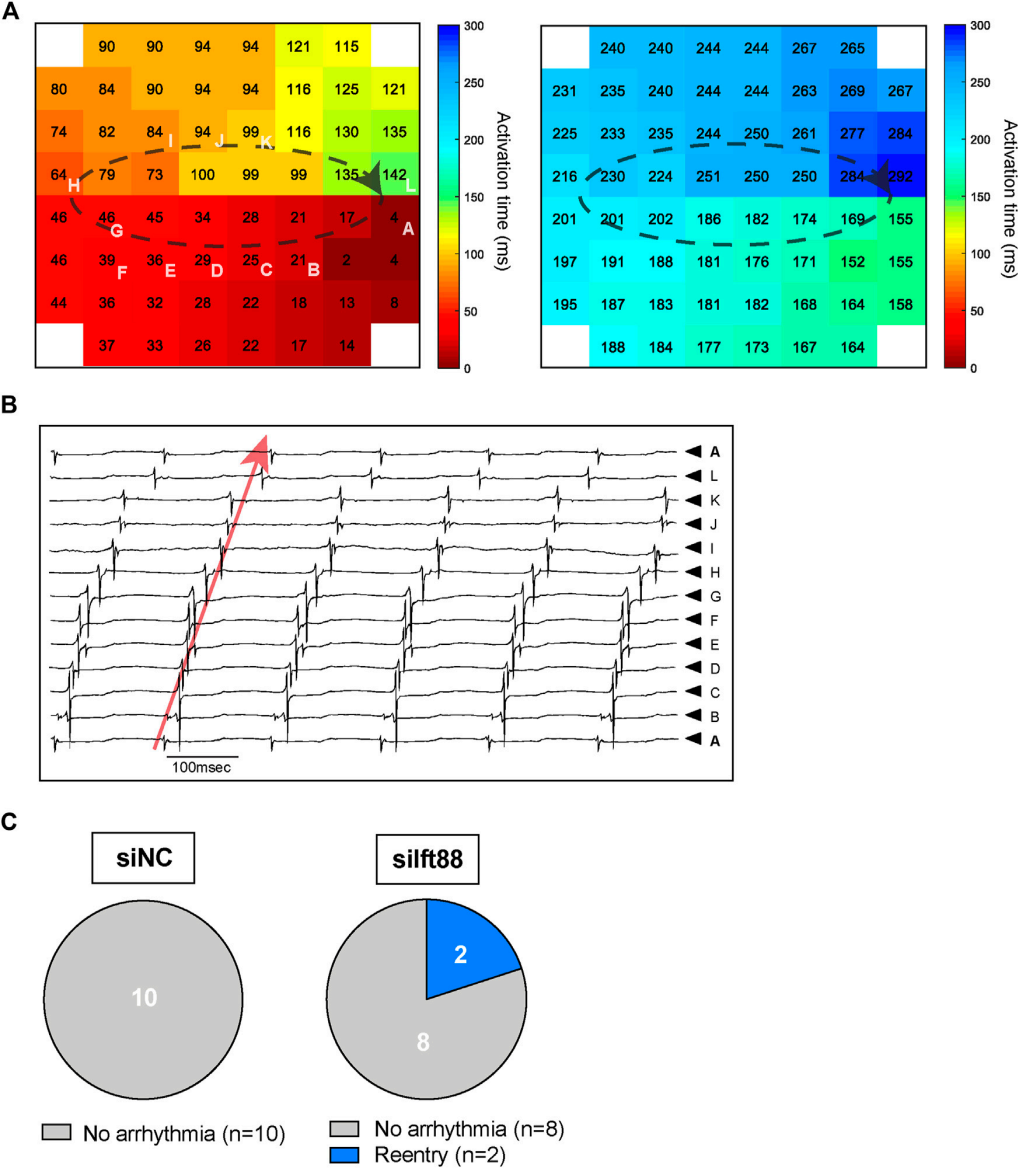
Reentry in CM co-cultured with siIft88 FB. **(A)** Activation maps of two following beats showing reentrant circuit and obtained from one CM monolayer co-cultured for 48 h with FB previously transfected with siIft88. Colors indicate activation times, according to the scale at right. Dashed black line indicated reentrant conduction pattern. Letters indicate electrodes from which selected unipolar electrograms are depicted in **(B)**. **(B)** Electrograms selected along the reentry circuit. Red arrow shows that the diastolic interval is spanned within local activation (electrogram from electrode A is repeated on top and at the bottom) as expected during reentry. The letters next to the electrograms indicate electrodes from which selected unipolar electrograms are shown **(A)**. **(C)** Proportion of CM-FB co-cultures showing reentrant arrhythmia or no arrhythmia in the siNC and siIft88 groups.

Figure 3A shows example representative activation maps of a spontaneous rhythm in one MEA in the siIft88 group. Activation times (small numbers) in both panels indicate activation times relative to the same reference time. The white letters indicate electrodes from which selected unipolar electrograms are depicted in panel B. The activation maps show two cycles with reentrant activation patterns in one CM-FB co-culture from the siIft88 group. Activation is continuous from the last activated site in the left panel to the first activated site in the right panel. Electrograms selected along the reentrant circuit are shown in Figure 3B and show that the diastolic interval is spanned by local activation, as expected during reentry. Overall, we observed two spontaneous reentrant activation patterns in two different CM-FB co-cultures in the siIft88 group and none in the siNC group (Figure 3C).

### Knockdown of Ift88 in fibroblasts is not associated with changes of action potential characteristics nor reduced electrical coupling

**FIGURE 4:**
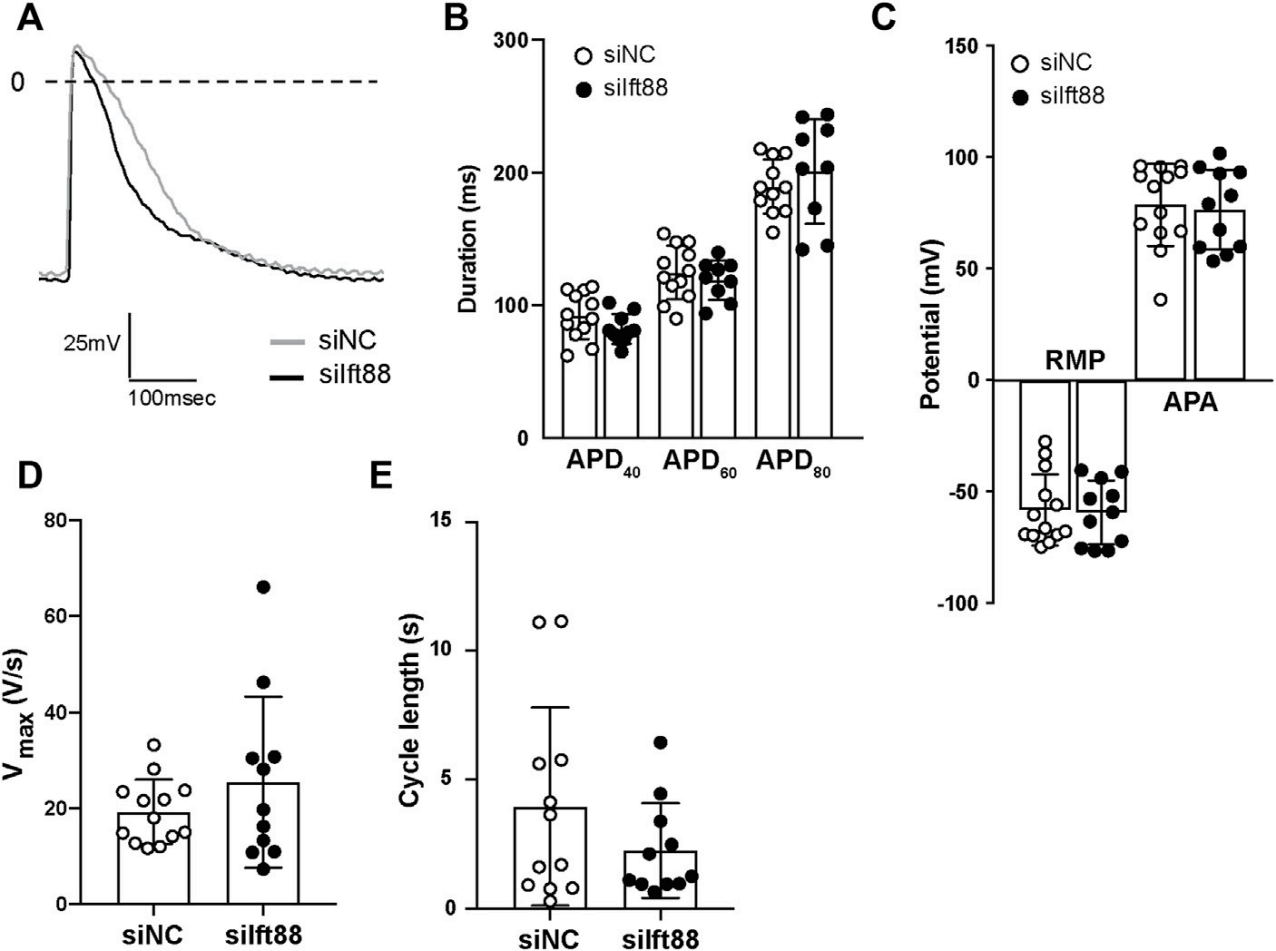
Co-culture between FB with reduced (mRNA) *Ift88* expression and CM does not lead to changes of the action potential characteristics. **(A)** Representative spontaneous APs recorded in CM monolayers co-cultured for 48 h with FB previously transfected with siNC or siIft88. **(B–E)** Effect of co-culture on spontaneous APs duration at 40%, 60%, and 80% (APD_40_, APD_60_, and APD_80_) of repolarization **(B)**, resting membrane potential (RMP), AP amplitude (APA) **(C)**, maximal upstroke velocity (V_max_) **(D)** and cycle length **(E)**. *n* ≥ 9 microelectrode measurements from two independent CM/FB isolation. Data are mean ± SD.

Figure 4A shows representative examples of microelectrode AP measurements in CM-FB co-cultures from siNC and siIft88 groups. Overall, there were no significant differences in APD_40_ [92.03 ± 17.60 (siNC) vs. 82.24 ± 11.25 ms (siIft88)], APD_60_ [124.6 ± 20.21 (siNC) vs. 119.1 ± 14.83 ms (siIft88)], nor in APD_80_ [189.4 ± 20.54 (siNC) vs. 201.1 ± 39.50 ms (siIft88)] between siNC and siIft88 co-cultures (*n* ≥ 9) (Figure 4B).

The observed reduction in conduction velocity (Figure 2) was not associated with significant depolarization of CM nor reduction of AP amplitude. Overall, the RMP was −57.96 ± 15.92 mV in the siNC co-cultures, while it was −59.23 ± 14.30 mV in the siIft88 co-cultures (*n* ≥ 9). The averaged AP amplitude was 78.94 ± 18.52 mV in the siNC co-cultures while it was 76.73 ± 17.83 mV in the siIft88 co-cultures (Figure 4C). V_max_ and cycle length were also not significantly different between the two groups [19.24 ± 6.719 (siNC) vs. 25.43 ± 17.79 V/s (siIft88) and 3.96 ± 3.84 (siNC) vs. 2.25 ± 1.84 s (siIft88), respectively] (Figures 4D,E).

Connexin43 (main protein component of gap junctions in neonatal rat ventricular CM) expression and distribution were not different between siNC and siIft88 co-cultures (Supplementary Figures S3, S4). Finally, the expression of major cardiomyocytes ion channels (*Kcnj11*, *Kcnq1*, *Kcnj2*, *Cacna1g*, *Scn1b*, *Scn5a*) was not significantly different between siIft88 and siNC groups (Supplementary Figure S5).

## Discussion

In this study, we show that the disruption of cilia formation in FB *via* targeting *Ift88* with siRNA is associated with enhanced ECM gene expression and conduction delay in CM-FB co-cultures, without changes of AP characteristics.

The cardiac ECM is a dynamic and complex network which plays an important role in providing structural support to cardiac cells, and regulates gene expression as well as gap junction organization (Forte et al., 2012). Remodeling of the ECM under pathological conditions alters cardiac fibers organization, leading to heterogeneous conduction delay and enhanced anisotropy, increasing the risk of arrhythmias.

Dysregulation of primary cilia in cardiac diseases and fibrosis formation has been implicated in various studies. For instance, primary cilia are important for embryonic development (Komatsu and Mishina, 2013) and the patients affected by a ciliopathy, a genetic condition caused by dysfunction of primary cilia, often manifest congenital heart disease and multi-organ fibrosis (Seeger-Nukpezah and Golemis, 2012; Klena et al., 2017). Several age-dependent diseases, such as atherosclerosis was shown to be driven by loss of primary cilia in aortic endothelial cells (Dinsmore and Reiter, 2016), suggesting that primary cilia suppress pro-atherosclerotic signaling. Biological processes underlying atrial fibrosis formation, such as epithelial-mesenchymal transition, differentiation of fibroblasts into myofibroblasts, and ECM synthesis depend on primary cilia (Ten Dijke et al., 2012).

Co-culture of CM and FB with a reduction of primary cilia by knockdown of lft88 leads to increased ECM gene expression of *Fn1*, *Col1a1* and *Ctgf* (Figure 1). This is in accordance with previous work showing that conditional knockout of *Ift88* in mouse heart leads to increased production of ECM substrates collagen I and versican (Toomer et al., 2017). Consistently, silencing *Ift88* in chondrocytes leads to increased expression of ECM components *Mmp13*, *Adamts5*, *ColX*, and *Runx2* (Chang et al., 2012; Collins and Wann, 2020). These results suggest that primary cilia are dynamic cellular appendages which restrain and fine-tune FB activation and ECM production. Consistent with these previous reports, we previously observed that FB from patients with persistent AF express fewer primary cilia in association with more ECM production compared to FB from non-AF patients (data not shown).

Our results are in seemingly contradiction with the study by Villalobos et al. where the conditional knockout of polycistine1 (PC1 encoded by pkd1, pkd1-cKO) in activated cardiac fibroblasts reduces scar size in a myocardial infarction (MI) model. However, this reduction was not attributable to reduced fibrosis. PC1 together with polycistine2 (PC2, encoded by pkd2) form a transient receptor potential ion channel and mediate the mechano-sensation *via* primary cilia, but they are not involved in the formation of primary cilia (Surya M. Nauli et al. Nature Genetics, 2003). Therefore, from the paper by Villalobos et al. it remains unclear whether a loss of primary cilia in the activated cardiac fibroblasts increases or decreases cardiac fibrosis.

We observed differences in *Ift88* transcript levels between FB cultures and CM-FB co-cultures (Figure 1). We speculate that these differences are caused by *Ift88* expression in cardiomyocytes in which *Ift88* was not knocked-down.

Co-culture of CM with FB transfected with siIft88 led to conduction abnormalities (slower CV, increased number of block lines) (Figure 2) and subsequent increased risk of reentrant arrhythmias (Figure 3), without changes in AP characteristics (Figure 4) and without reduction in *Gja1* expression (Supplementary Figure S1). This demonstrates that the observed CV reduction and increased risk of arrhythmia is not subsequent to electrical remodeling of CM (i.e., reduction of intercellular electrical coupling between CM or depolarization of CM). Instead, the CV reduction and increased number of block lines observed in the siIft88 CM-FB co-cultures is likely due to the increased ECM production from activated fibroblasts following the dysregulation of primary cilia. Excessive ECM deposition separates CM and may lead to tortuous patterns of activation which results in conduction delay and blocks. These findings are in accordance with literature showing that conduction velocity is impaired when (myo)fibroblast concentration increases and fibrosis occurs (Spencer et al., 2017).

## Study limitations

While knockdown of IFT88 is known to cause loss of primary cilia in fibroblasts, we cannot exclude that there are other, secondary, effects caused by this gene knockdown which could modify fibroblast behavior.

Extracellular matrix remodeling was in this study assessed by gene expression measurement, without quantifying the ECM architecture or composition. Further study should investigate and perform quantitative analysis on the ECM architecture and composition in the two groups.

## Conclusion

Protecting the function of primary cilia and repairing the disrupted cilia is a potential therapy to prevent the onset of AF and to reverse the fibrosis formation in the advanced AF, respectively.

## Data Availability

The raw data supporting the conclusion of this article will be made available by the authors, without undue reservation.
