# Supplementary material for: Knockdown of Ift88 in fibroblasts causes extracellular matrix remodeling and decreases conduction velocity in cardiomyocyte monolayers
Source: Front Physiol. 2022 Nov 17;13:1057200. doi: 10.3389/fphys.2022.1057200 (PMC9713696; doi:10.3389/fphys.2022.1057200)
Supplement: Supplementary file 1 [file DataSheet1.docx]

Supplementary Material


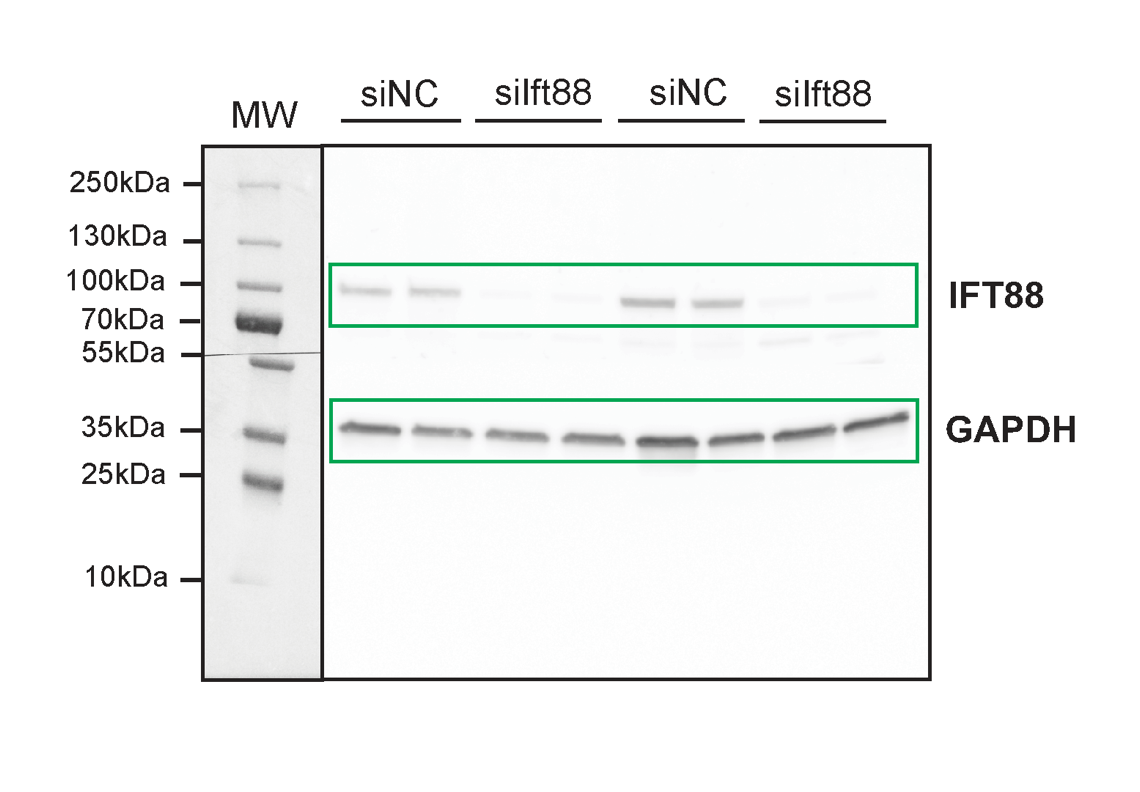


**Supplementary Figure 1. Full unedited Western blot for Figure 1.**

Full unedited Western blot for Figure 1 of IFT88 protein quantification in fibroblasts from siNC and siIft88 groups.


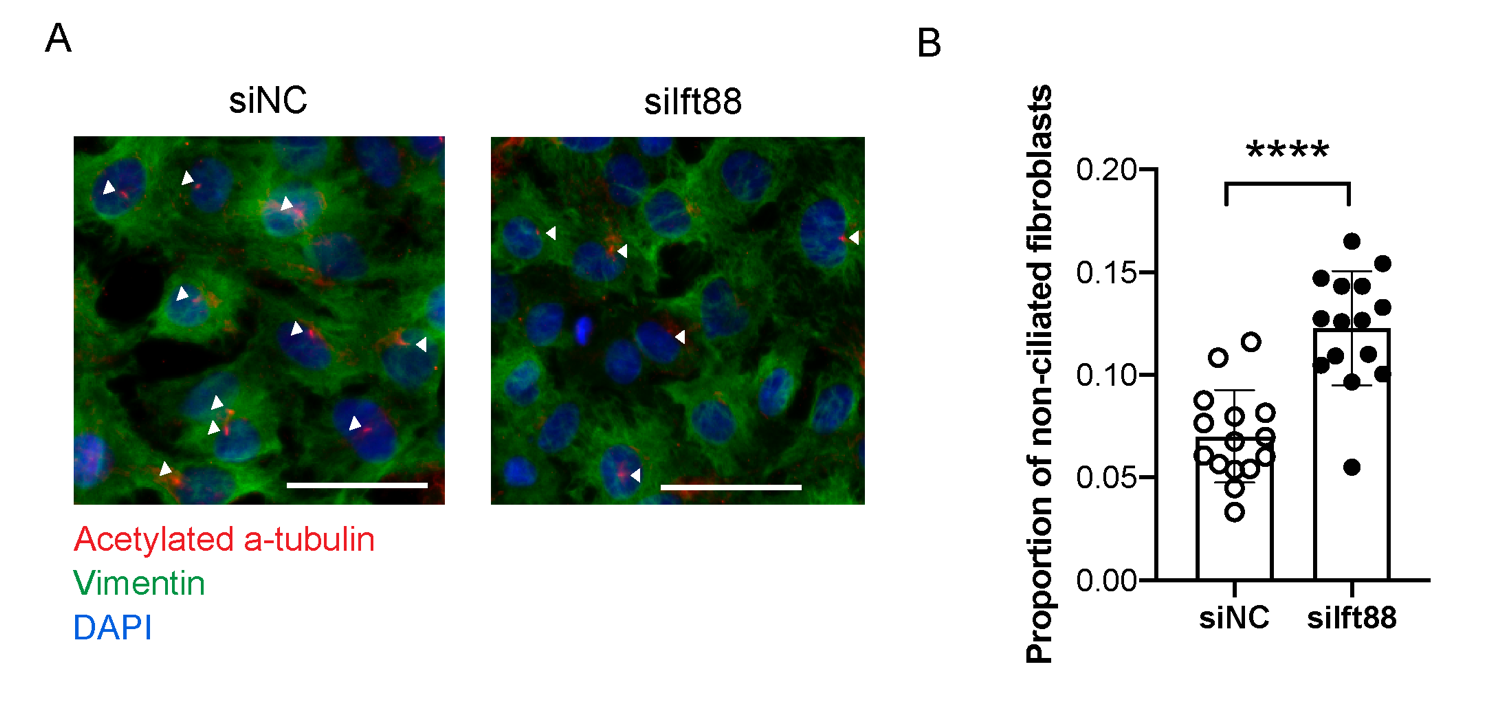


**Supplementary Figure 2. Proportion of non-ciliated fibroblasts after *Ift88* knockdown**

(A) Immunofluorescence images of fibroblasts from siNC and siIft88 groups stained with acetylated α-tubulin (red), vimentin (green), DAPI (blue). Antibodies references can be found in Table 2. Scale bar, 50µm.

(B) Proportion of non-ciliated fibroblasts in siNC and siIft88 groups. Data are mean±SD. n=3 coverslips were imaged per group, taking 5 images per coverslips. Student's t-test.

****p<0.0001


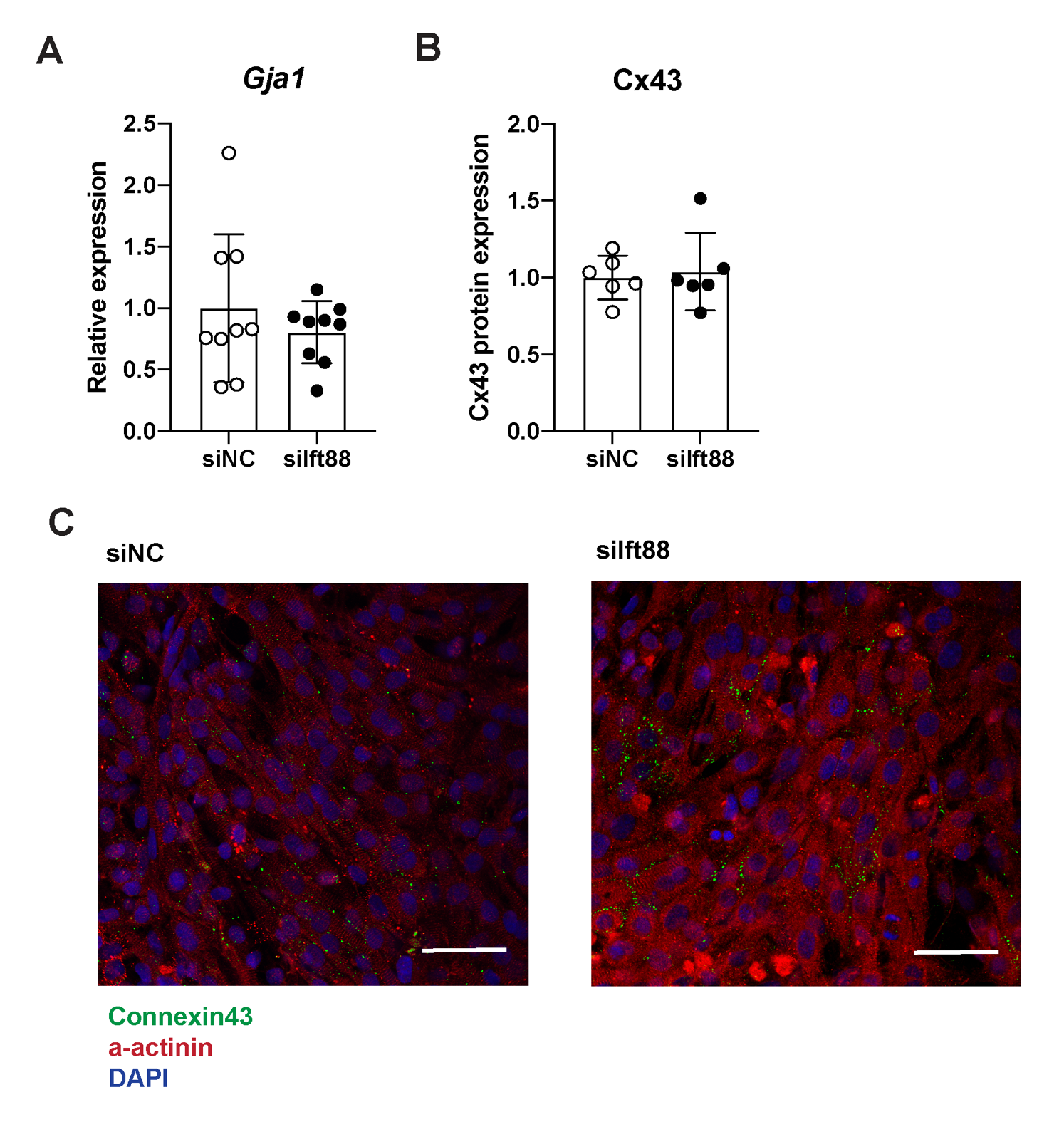


**Supplementary Figure 3. *Gja1* expression in CM-FB co-cultures.**

(A) Relative expression of *Gja1*, gene encoding for Connexin43, in CM-FB co-cultures from siNC and siIft88 groups, measured by real-time qPCR. Data are mean±SD. n=9 from three independent CM-FB isolation.

(B) Relative protein expression of Cx43 in CM-FB co-cultures from siNC and siIft88 groups, measured by Simple Wes. Cx43 signal was normalized to calnexin. Data are mean±SD. n=6 from two independent CM-FB isolation. Full blots are provided in Supplementary Figure 4.

(C) Immunofluorescence images of co-cultures from siNC and siIft88 groups stained with CX43 (green), α-actinin (red), DAPI (blue). Antibody references can be found in Table 2. Scale bar, 50µm.


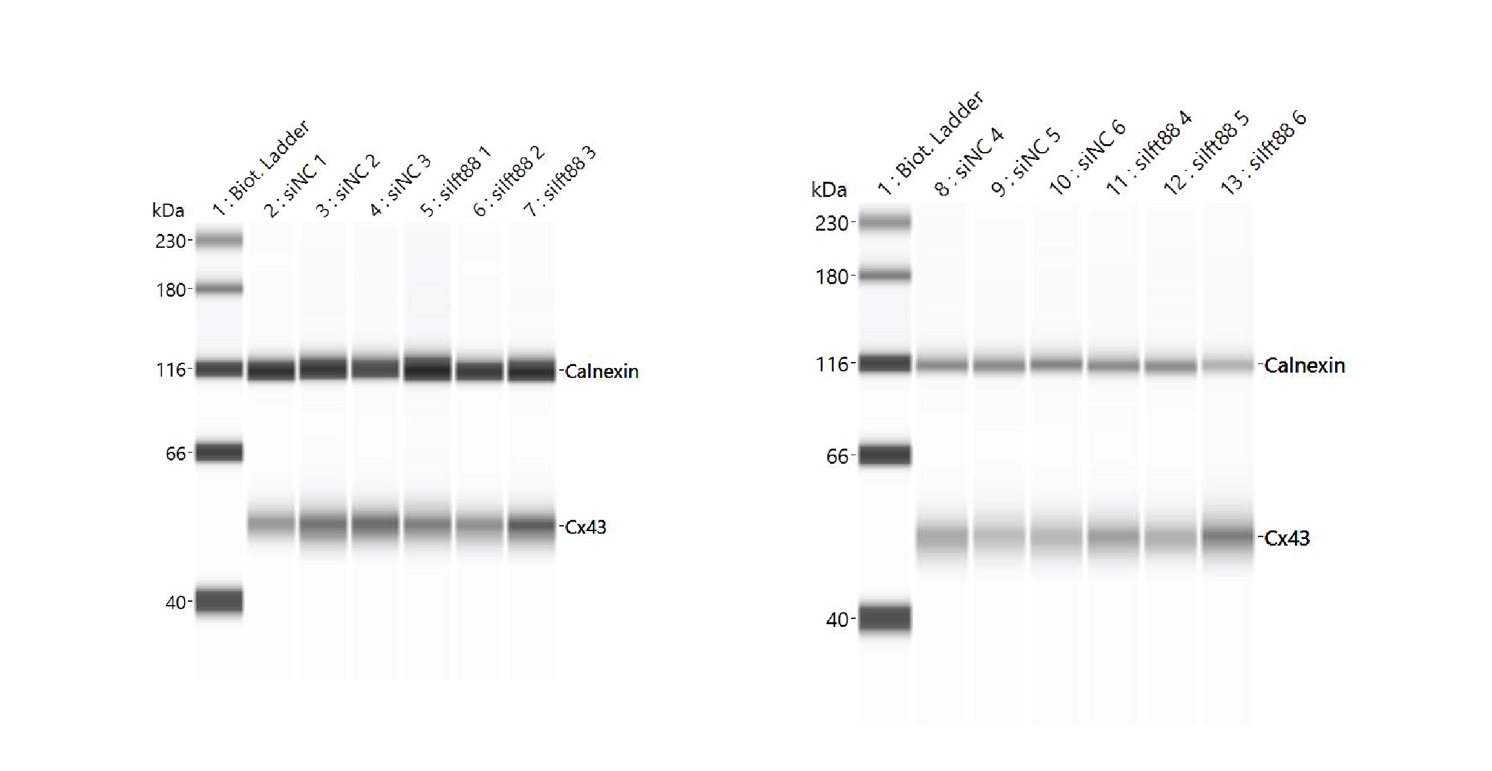


**Supplementary Figure 4. Full unedited Simple Wes blots.**

Full Simple-WES blots of Connexin 43 protein quantification in CM-FB co-cultures from siNC and siIft88 groups.

**
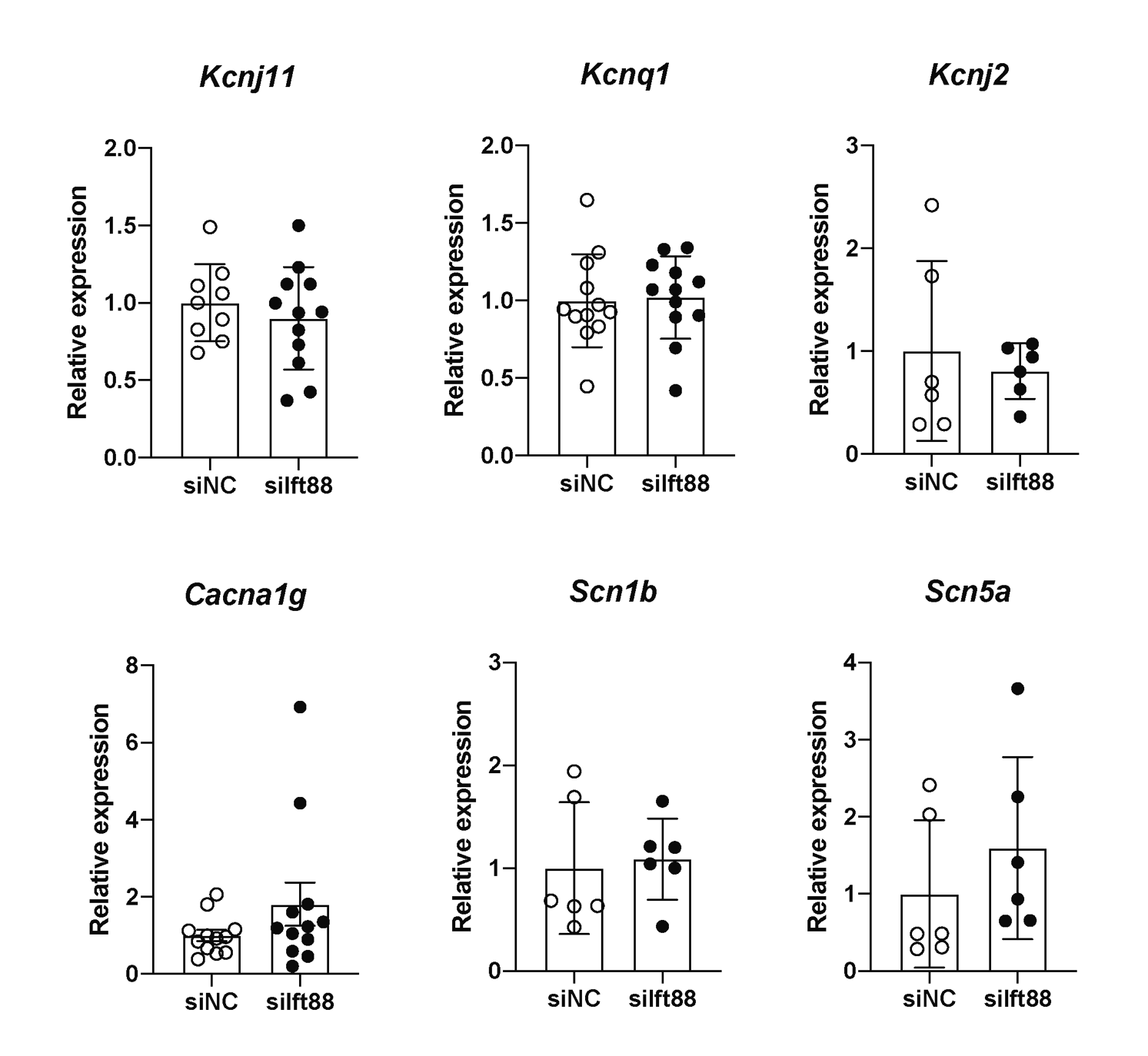
**

**Supplementary Figure 5.**

mRNA levels expression of potassium, calcium and sodium pumps related genes in CM-FB co-cultures. Data are mean±SD. n=6 minimum from two independent CM-FB isolation minimum.
